# Fatty acid desaturase 2 (FADS 2) rs174575 (C/G) polymorphism, circulating lipid levels and susceptibility to type-2 diabetes mellitus

**DOI:** 10.1038/s41598-021-92572-7

**Published:** 2021-06-23

**Authors:** Shilpa S. Shetty, N. Suchetha Kumari

**Affiliations:** 1 Central Research Laboratory, K.S.Hegde Medical Academy, Nitte (Deemed To Be University), Deralakatte, Mangalore, India; 2 Department of Biochemistry, K.S.Hegde Medical Academy, Nitte (Deemed To Be University), Deralakatte, Mangalore, India

**Keywords:** Biochemistry, Genetics, Molecular biology, Diseases, Endocrinology, Health care, Medical research

## Abstract

Several factors influence an individual’s susceptibility in inter-individual lipid changes and its role in the onset of type-2 diabetes mellitus (T2DM). Considering the above fact, the present investigation focuses on determining the association between fatty acid desaturase 2 (FADS2) rs174575 (C/G) polymorphism, circulating lipid levels and susceptibility to type-2 diabetes mellitus. As per the inclusion and exclusion criteria a total of 429 subjects (non-diabetic-216; diabetic-213) were recruited for the study. Glycemic and lipid profile status were assessed using commercially available kits. Based on the previous reports SNP rs174575 of fatty acid desaturase gene (FADS2) was selected and identified using the dbSNP database. The amplified products were sequenced by means of Sanger sequencing method. Lipid profile status and apolipoprotein levels revealed statistically significant difference between the groups. Three models were assessed namely, recessive model (CC vs CG + GG), dominant model (CC + CG vs GG) and additive model (CC vs CG vs GG). The recessive model, displayed a statistically significant variations between the circulating lipid levels in T2DM. The multivariate model with genotype (G allele carriers), triglyceride (TG) and insulin served as a predictive model. The study results potentiate the functional link between FADS2 gene polymorphism, lipid levels and type-2 diabetes mellitus.

## Introduction

Type 2 diabetes mellitus (T2DM) is referred as an epidemic, increasing at an alarming rate. All around the world, 425 million people present diabetes, and it is estimated to be 629 million in 2045 (IDF Atlas)^1^, including both diagnosed and undiagnosed diabetes. Among the diabetic cases around the globe India secured top position with more than 32 million diabetics, and the number is anticipated to increase up to 79.4 million by 2030^2^.

Higher prevalence of diabetes is seen in India when compared to western countries despite lower overweight and obesity rates, suggesting occurrence of diabetes in patients with lower body mass index (BMI)^3,4^^.^ Hence Indian adults with comparatively lower BMI also poses equal risk as individuals who are obese^5,6^. Joshi SR reported that Asian Indians consists of smaller body size but suffer from central obesity (higher waist to hip ratio) and higher subscapular-to-triceps skin fold ratio when compared with their British counterparts, hence the term thin-fat Indian^7^. Furthermore, it also reflected in triacylglycerol (TG) and higher plasma non-esterified fatty acid (NEFA) concentrations, hyperinsulinemia (with fasting), higher insulin resistance (IR) and post-glucose challenge states^7,8^. Henceforth, in Asian Indians, insulin resistance (IR) is associated with thin-fat unusual body composition^7,9^.

A complicated association exists between type 2 diabetes and dyslipidemia^10,11^ with majority of study revealing insulin resistance primary to varied lipid concentrations^12^, but few studies illustrated that dyslipidemia could be a contributing factor for the pathogenesis of type 2 diabetes^13^ by impairing ß-cell protection or endoplasmic reticulum stress (ER Stress)^14,15^. Complex interactions between nutrients and genes modulate an individual’s risk for disease development^16^. Multiple factors governs an individual’s lipid levels and their inter-individual differences. The contribution of genetic variability to these differences are still unanswered. Therefore, identification of lipid modulating gene variants is critical for understanding the disease pathogenesis. Previous studies have demonstrated the role of fatty acid desaturases in determining the plasma and tissue fatty acid profiles elucidating various molecular pathways of lipid metabolism^17,18^. Further, correlation between fatty acid desaturase (FADS) genetic variants (encoding rate-limiting enzymes for PUFA synthesis) with desaturase activities and blood lipids are also reported^19^. When considering these results it can be concluded that the desaturation pathway appears to be extremely important for lipid homeostasis in the human body. There exist a research gap to explore whether genetic variations of FADS2 would exert effects on circulating lipid levels in type-2 diabetes mellitus^19,20^. The present study aims to find an association between fatty acid desaturase 2 gene polymorphism and circulating lipid levels in type-2 diabetes.

## Materials and methods

The Central Ethics committee of Nitte (Deemed to be University) reviewed and approved the study for human subjects. Informed consent was duly signed after procuring institutional ethical clearance. Subjects were recruited (Non-diabetic-216; Diabetic-213) based on the inclusion and exclusion criteria. The Subjects were recruited according to the ADA criteria^21^. Subjects under the age group of 30-60yrs, both sexes, diagnosed with T2DM atleast 3 months prior screening were included in the study group. All methods were performed in accordance with the relevant guidelines and regulations.

### Anthropometric measurements and biochemical estimations

Fasting plasma glucose (FBS), Glycated hemoglobin (HbA1c), and plasma insulin were measured as described previously^22^. Lipid profile status (TC, TG, HDL-C) were analysed using commercially available kits (LiquiCHEK^TM^AGAPPE). Friedewald formula was used for Low-Density LDL cholesterol (LDL-C) calculation^23^, in subjects with serum TG concentrations < 400 mg/dL (4.52 mol/L). Subjects with serum TG concentrations of ≥ 400 mg/dL were excluded for LDL-C calculation. ApoA1 and apo B100 were estimated using a commercially available kit (Agape SensIT).

### Isolation, quantification of DNA and SNP selection

DNA was isolated using a standardized protocol by Sergeant et al.^24^_._ The concentration of DNA per sample was determined using bio-spectrophotometer and stored at − 20 °C.

The SNP rs174575 of FADS2 was selected according to previous literature^25–28^ and identified using the dbSNP database^29^ based on the National Centre for Biotechnology Information (NCBI)^28^. The primer pair for SNP rs174575 (designed for the study) involved in the study was as follows: Forward: AGGCAGATGGACCTGGATTTGA and Reverse TGGCTTGCAAATAGACTCATCTCC. PCR and Sanger sequencing was performed as described previously^22^.

### Statistical analysis

SPSS (version 20.0, IBM, Armonk, NY, USA) was used for statistical analysis. The variable between different genotypes were analysed by One-way ANOVA and Bonferroni correction tests. Logistic regression analysis was performed. The discriminative ability of a substantial model with the highest Odds risk (OR) was analysed by receiver operating characteristic (ROC) analysis. *p* value < 0.05 was considered to be statistically significant.

## Results

### General, anthropometric and biochemical characteristics of the study population

The anthropometric, demographics and glycemic profile of the study population is described previously and has shown a statistically significant difference (*p* < 0.001)^22^.

Among the diabetic group, 93% of the subjects presented dyslipidemia. Further the pattern of dyslipidemia was studied which displayed that 47.9% combined type of dyslipidemia (low HDL-C and high TG, high LDL-C and high TG, low HDL-C and high LDL-C), 16.4% revealed mixed (high LDL-C, TG and low HDL-C) and 28.6% exposed single type (high LDL-C, high TG and low HDL-C) of dyslipidemia pattern. From the measurements it was noticed that LDL-C (74.20%) was the most deranged lipid type in the diabetic subject in the present study.

Apo-A1 was lower and ApoB100 levels were higher respectively in the diabetic group and illustrated a high statistical significance (*p* < 0.001) (Table 1). On comparing the apolipoprotein ratio (apoB100/apoA1), the diabetic group showed a higher value than non-diabetic group with high statistical significance.

**Table 1:** Comparison of the mean lipid profile and apolipoprotein levels in diabetic and non- diabetic individuals.

Parameters (mg/dl)	Non-diabetic (N = 216)	Diabetic (N = 213)	*p* value
TC*	164.78 ± 37.99	211.46 ± 51.29	< 0.001
TG^✥^	120.25 (88.92–161.95)	162.00 (124.9–218.45)	< 0.001
HDL-C*	44.14 ± 19.3	43.38 ± 15.49	0.786
LDL-C*	93.71 ± 43.19	127.84 ± 48.08	< 0.001
VLDL^✥^	24.05 (17.784–32.39)	32.00(24.5–42.28)	< 0.001
Apo A	129.353 ± 6.064	124.962 ± 4.466	< 0.001
ApoB100	76.401 ± 1.993	87.340 ± 4.397	< 0.001
Apo B100/Apo A	0.592 ± .035	0.700 ± 0.046	< 0.001

^✥^*P* value ≤ 0.05 was considered statistically significant.

*Student t-test, Data are shown as mean ± SD.

^✣^Mann–Whitney *U* test. Data are shown as median (interquartile range).

### Correlation between glycemic profile, lipid profile and apolipoproteins in T2DM

The glycemic profile showed positive correlation with lipid profile and apoliporotein status except for HOMA-B and apoA1 (Table 2).

**Table 2:** Correlation between glycemic profile, lipid profile and apolipoproteins in type 2 diabetes mellitus.

		HbA1C	TC	TG	HDL-C	LDL-C	VLDL	Insulin	HOMA-IR	HOMA-B	Apo A	Apo B100	Apo B100/ Apo A1
**FBS (mg/dl)**	Correlation	0.71	0.33	0.23	− 0.09	0.267	0.193	0.56	0.974	− 0.562	− 0.324	0.588	0.582
	Significance (2-tailed)	< 0.001	< 0.001	0.072	< 0.001	< 0.001	< 0.001	< 0.001	< 0.001	< 0.001	< 0.001	< 0.001	< 0.001
**HbA1C (%)**	Correlation		0.39	0.23	− 0.06	0.32	0.179	0.515	0.713	− 0.435	− 0.261	0.532	0.511
	Significance (2-tailed)		< 0.001	0.235	< 0.001	< 0.001	< 0.001	< 0.001	< 0.001	< 0.001	< 0.001	< 0.001	< 0.001
**TC (mg/dl)**	Correlation			0.352	0.052	0.867	0.341	0.424	0.374	− 0.256	− 0.204	0.386	0.378
	Significance (2-tailed)			0.297	< 0.001	< 0.001	< 0.001	< 0.001	< 0.001	< 0.001	< 0.001	< 0.001	< 0.001
**TG (mg/dl)**	Correlation				− 0.015	− 0.07	0.858	0.257	0.256	− 0.201	− 0.031	0.236	0.185
	Significance (2-tailed)				0.167	< 0.001	< 0.001	< 0.001	< 0.001	0.531	< 0.001	< 0.001	< 0.001
**HDL-C (mg/dl)**	Correlation					− 0.24	0.029	− 0.09	− 0.094	0.156	− 0.027	− 0.06	− 0.035
	Significance (2-tailed)					0.567	< 0.001	0.082	0.002	0.594	0.201	0.489	0.489
**LDL-C (mg/dl)**	Correlation						− 0.03	0.353	0.303	− 0.223	− 0.19	0.316	0.321
	Significance (2-tailed)						< 0.001	< 0.001	< 0.001	< 0.001	< 0.001	< 0.001	< 0.001
**VLDL**	Correlation							0.24	0.219	− 0.187	− 0.043	0.215	0.174
	Significance (2-tailed)							< 0.001	< 0.001	0.394	< 0.001	< 0.001	< 0.001
**Insulin pmol/l**	Correlation								0.719	− 0.335	− 0.292	0.716	0.657
	Significance (2-tailed)								< 0.001	< 0.001	< 0.001	< 0.001	< 0.001
**HOMA-IR**	Correlation									− 0.533	− 0.333	0.66	0.641
	Significance (2-tailed)									< 0.001	< 0.001	< 0.001	< 0.001
**HOMA-B**	Correlation										0.215	− 0.43	− 0.42
	Significance (2-tailed)										< 0.001	< 0.001	< 0.001
**Apo A**	Correlation											− 0.36	− 0.714
	Significance (2-tailed)											< 0.001	< 0.001

*P value ≤ 0.05 was considered statistically significant.

### Comparison of the lipid profile in diabetic individuals in different genotype models

CC genotype was wild type homozygote genotype for rs174575 C > G, while GG was homozygous recessive variant. The allele and genotype frequencies have been described previously^22^. Three models were included in this study—Additive model (CC vs CG vs GG); Dominant model (CC + CG vs GG); Recessive model (CC vs CG + GG) to compare the lipid profile and apolipoprotein levels status with the genotype.

In the additive model, TC, LDL-C and VLDL-C revealed statistically significant difference (Table 3). TC, LDL-C, TG and VLDL revealed statistically significant difference between the groups in the recessive model (Table 3). No statistically significant difference was observed in the dominant model (Table 3).

**Table 3:** Comparison of lipid profile and apolipoprotein levels between the diabetic individuals using different genotype models.

Parameters (mg/dl)	Additive model (CC vs CG vs GG)				Dominant model (CC + CG vs GG)			Recessive model (CC + CG vs GG)		
	CC (N = 180)	CG (N = 29)	GG (N = 4)	*p* value	CC + CG (N = 209)	GG (N = 4)	*p* value	CC (N = 180)	CG + GG (N = 33)	*p* value
**TC**	206.84 ± 3.73	235.50 ± 9.35	244.99 ± 25.02	0.008	210.67 ± 3.48	252.61 ± 25.59	0.187	206.99 ± 3.69	235.78 ± 8.68	0.002
**TG**	188.97 ± 9.50	225.59 ± 23.82	183.39 ± 63.73	0.107	193.99 ± 8.88	186.46 ± 65.33	0.899	189.46 ± 9.55	217.84 ± 22.44	0.041
**HDL-C**	45.07 ± 1.44	43.81 ± 3.61	42.20 ± 9.67	0.914	44.84 ± 1.32	44.79 ± 9.71	0.784	44.92 ± 1.42	44.39 ± 3.35	0.693
**LDL-C**	123.98 ± 3.54	146.58 ± 8.89	166.11 ± 23.77	0.018	127.03 ± 3.29	170.52 ± 24.20	0.111	124.18 ± 3.52	147.82 ± 8.27	0.006
**VLDL**	36.23 ± 1.72	44.89 ± 4.32	27.38 ± 11.56	0.043	37.41 ± 1.62	28.49 ± 11.94	0.535	36.23 ± 1.74	42.78 ± 4.09	0.036
**Apo A**	124.86 ± 0.34	125.26 ± 0.84	127.34 ± 2.25	0.514	124.89 ± 0.31	128.37 ± 2.25	0.287	124.83 ± 0.33	125.66 ± 0.78	0.444
**Apo B100**	87.51 ± 0.33	86.59 ± 0.83	85.27 ± 2.21	0.381	87.38 ± 0.30	85.23 ± 2.24	0.346	87.49 ± 0.33	86.50 ± 0.77	0.203
**Apo B100/A1**	0.70 ± 0.003	0.69 ± 0.01	0.67 ± 0.02	0.250	0.70 ± 0.003	0.663 ± 0.024	0.191	0.70 ± 0.003	0.69 ± 0.01	0.162

*P value ≤ 0.05 was considered statistically significant.

### Genotype association (rs174575 (FADS2)) with T2DM

**Figure 1:**
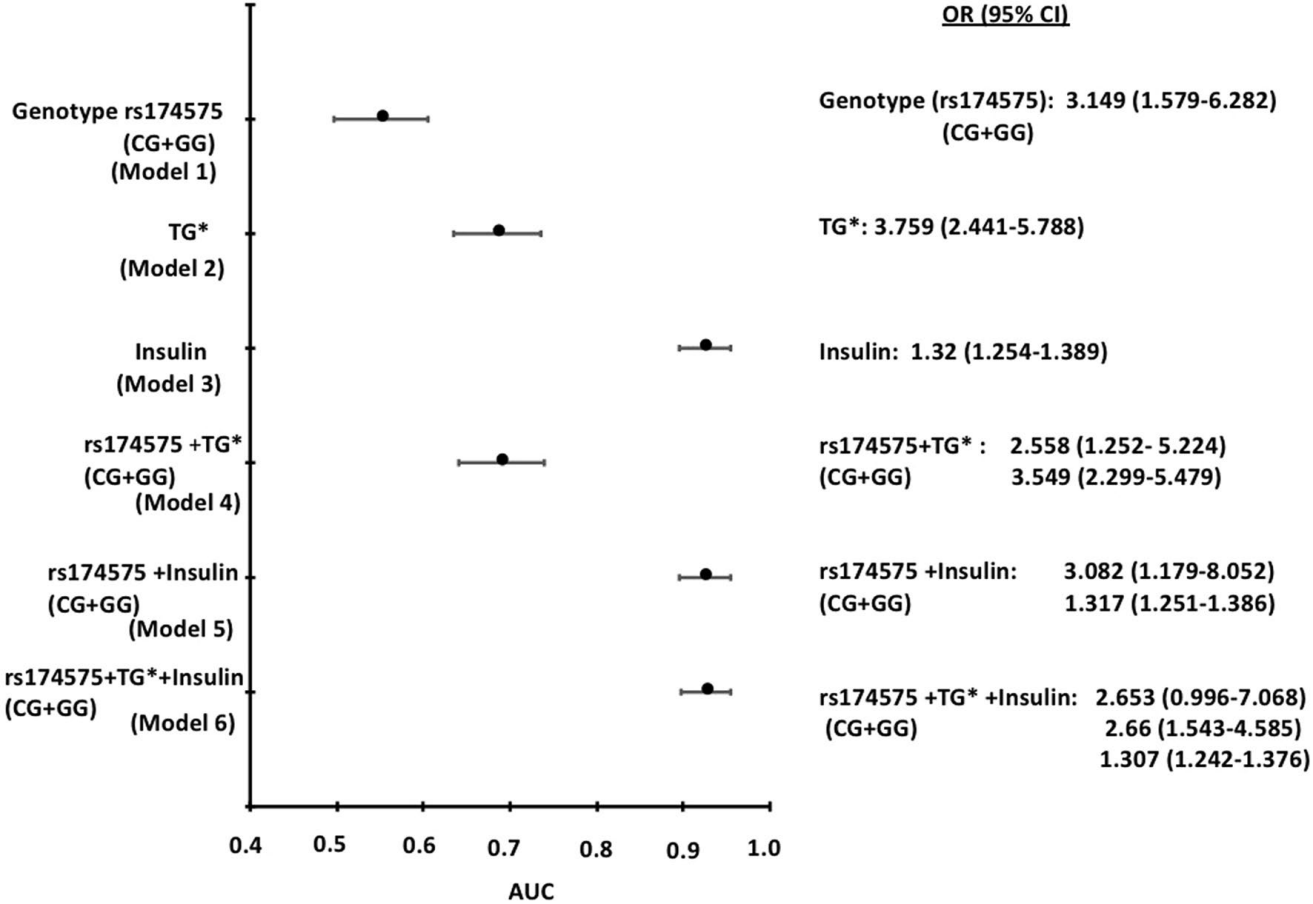
: Predictive models for association of FADS 2 (rs174575) and risk of T2DM.

To quantify the relationship between an exposure and a disease, association was measured as odds ratio. Logistic regression, univariate, bivariate and multivariate analysis were performed to identify whether there is any associations between the SNP’s , triglycerides, insulin and risk of T2DM. A total of six predictive models for genotype (CG + GG) were studied to assess the association of genotype with T2DM. Three univariate model: Genotype (CG + GG) (model 1), TG (model 2), Insulin (model 3); two bivariate model: genotype and TG (model 4), genotype and insulin (model 5) and one multivariate model including genotype, TG and insulin (model 6) (Fig. 1).

The results of univariate analysis showed that rs174575 of the FADS 2 gene showed an odds risk (OR) of 3.149 (95% CI 1.579–6.282) (Model 1), TG showed an odds risk (OR) of 3.759 (95% CI 2.441–5.788) (Model 2) and insulin showed an odds risk (OR) of 1.32 (95% CI 1.32 (1.254–1.389) (Model 3). In the bivariate model rs174575 and TG showed an OR of 2.558 (95% CI 1.252–5.224); 1.317 (95% CI 1.251–1.386) and rs174575 and insulin showed an OR of 3.082 (95% CI 1.179–8.052); 1.317 (95% CI 1.251–1.386). In the multivariate model rs174575, TG and insulin showed an OR of 2.653 (95% CI 0.996–7.068); 2.66 (95% CI 1.543–4.585); 1.307 (95% CI1.242–1.376).Area under the curve (AUC) (X-axis in Fig. 1) depicts the accuracy of the measurement of a test i.e., an area of 0.9–1 represents a perfect test; an area of 0.5–0.7 is no better than chance. Therefore, model 1 (AUC = 0.55), model 2 (AUC = 0.685) and model 4 (AUC = 690) showed AUC ≤ 0.7 therefore the association may be by chance. However, model 3 (AUC = 0.925), model 5 (AUC = 0.925) and model 6 (AUC = 0.926) showed and AUC > 0.9 suggesting perfect association with T2DM risk (Fig. 1).

## Discussion

Diabetic populations are also prone to lipid and lipoprotein abnormalities as an indirect effect of insulin resistance on key metabolic enzymes^30^. Effect of lipid levels with glucose levels is previously studied and is very well established^30,31^. Carbohydrates and lipid metabolism are interrelated to each other^30,31^. Our present investigation demonstrated significantly increased levels of total cholesterol (TC) in diabetic patients, with 56.4% having cholesterol levels above 200 mg/dl. This increase in TC can be due to the increased hepatic VLDL production or reduced removal of circulating VLDL and LDL-C^32^. It is evident from the present investigation that, VLDL and LDL-C increased in diabetic individuals. The diabetic subjects had a higher level of triglycerides (TG). About 58.7% of the diabetic subjects had triglyceride levels above 150 mg/dL. Elevated levels of triglycerides elevate free fatty acid level, which may in turn induce insulin resistance and β-cell dysfunction^31^. The elevated free fatty acid levels, in turn, impairs normal function of the β-cell by disrupting or modulating the cascade that links insulin receptors with glucose transporters^33^.

The study also showed a significantly increased level of LDL-C in diabetic patients. About 74.2% of the diabetic subjects had LDL-C levels more than 100 mg/dL of which 8.5% showed LDL levels of 190 mg/dL. LDL-C was the most prevalent type of lipid abnormality observed in the current study. This may be attributed to the higher insulin levels in the study. The number of LDL-C receptors depends on insulin and reduced levels of LDL-C receptors can be associated with chronic deficiency of insulin^34^. Therefore, in type-2 diabetic individuals, insulin increases in the bloodstream, due to insulin resistance, which in turn increases the LDL-C particles thereby increasing the LDL-C levels.

HDL-C pays a direct role in glucose metabolism. The study observed a decreased HDL-C levels in diabetic subjects than normal levels. TG enrichment by Cholesteryl ester transfer protein (CTEP) and increased hepatic triglyceride lipase activity, in turn, results in lowered HDL-C. The liver produces HDL-C particle. In the diabetic state, this metabolic pathway will be defective, hence limiting the HDL-C production from the source. Low HDL-C, low anti-inflammatory activity and reduced reverse cholesterol transport, altering microenvironment, increased insulin resistance and impaired β-cell function^34^.

Apo A1 is antiatherogenic^35^. In the present study, apoA1 was lower in diabetic this was not in accordance with the previous report^36^. Whereas apoB100 is atherogenic^37^. According to the previous studies, apo-B represents an ideal marker for the management of dyslipidemia in individuals with diabetes^38–40^. The apoB100/apoA1 ratio indicates antiatherogenic and atherogenic balance; the higher the value, higher the CV risk. On comparing the apolipoprotein ratio (apoB100/apo A1), the diabetic group showed a higher value than that of the non-diabetic group. The apoB100/apoA1 ratio is strongly associated with insulin resistance. In the current study, on analyzing the pattern of dyslipidemia in diabetic subjects, about 47.9% is due to the combined type of dyslipidemia followed by single and mixed type. Desaturation and elongation are steps of a metabolic pathway in which dietary and endogenous saturated fatty acids (SFAs) are lengthened and converted to mono-unsaturated fatty acids (MUFA), and highly polyunsaturated fatty acid (PUFA) are synthesized from dietary n-6 fatty acids (e.g., linoleic acid) and n-3 fatty acids (e.g., α-linolenic acid) in the liver and adipose tissue Fatty acid desaturase inset unsaturated bonds to fatty acid molecules. Previous studies have shown that even a single nucleotide polymorphisms (SNPs) in *FADS* genes are associated with not only altered fatty acid desaturase activity but has also shown profound changes in lipid profile status^41^. Allele frequencies and genotype frequencies are the two fundamental calculations of population genetics^42^. The minor allele frequency in the study subject was above 0.05 (i.e., 0.08 in non-diabetic and 0.09 in diabetic). All of the associated SNPs are intronic or intergenic variants, and there are scarce reports on exonic coding variants in the FADS genes^43^. The CC homozygote is the wildtype, CG is the heterozygote, and GG is the recessive variant. The frequency of occurrence of ‘G’ allele carriers were higher in diabetic individuals. Of the three genotype models deduced to study the relationship between genotype and the clinical characteristics, the recessive model (CC vs CG + GG) showed a statistically significant result. TC, LDL-C, TG and VLDL showed a statistical difference between the groups. From the study findings, the multivariate model with Genotype, TG and Insulin model showed an association between genotype and type-2 diabetes risk. Based on the odds risk values genotype itself showed a threefold higher association with diabetes i.e., the minor G allele carriers are the risk group. In the present study since the population size is small, insulin and TG add value to the model. Based on the study results a suggestive mechanism elucidating the association between FADS 2 gene polymorphism and lipid levels and type-2 diabetes susceptibility is as follows: FADS 2 gene encodes for the enzyme delta-6 desaturase which is a rate-limiting enzyme in polyunsaturated fatty acids (PUFA) metabolism. FADS 2 minor alleles were associated with decrease enzyme activity and therefore less conversion of precursors to products^44^.

**Figure 2:**
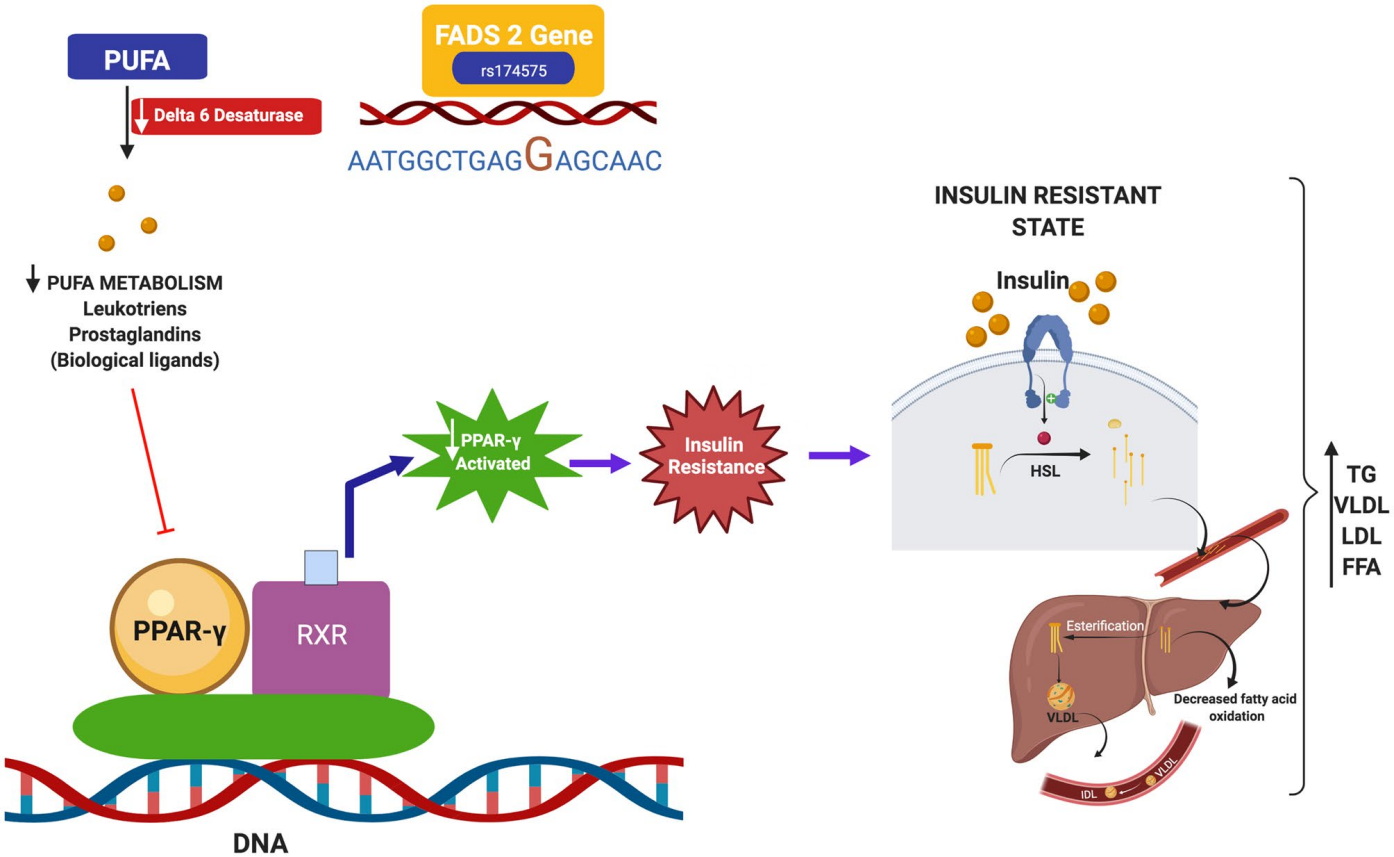
Proposed mechanism showing association between FADS 2 gene polymorphism, lipid levels and type-2 diabetes mellitus.(Created with BioRender.com)

From the literature it is evident that FADS 2 SNPs interact with genes such as PPAR-γ which in turn is closely associated with IR, Mets and DM^45^. PPAR-γ acts as a dominant regulator of adipogenesis and plays an important role in lipogenesis^45^. Ligands of PPAR-γ may be biological or synthetic. The synthetic ligands include thiazolidinedion (TZD) (anti-diabetic drug) and fibrates (hyperlipidimic) whereas biological ligands are PUFA’s, prostanoids, leukotrienes etc. From this a suggestive mechanism of action of minor allele carriers as derived from the study is that the minor allele carriers show altered PUFA metabolism wih omega 3 fatty acids and increased LA (from dietary major sources such as seed oils) and AA, accumulation, activates NF-Kappa B, transcribing COX and lipo-oxygenese genes and increasing inflammation, reduce insulin sensitivity and increased insulin resistance which in turn results in increasing HSL activity which hydrolysis TG to glycerol and free fatty acids (FFA) which in turn is released to the circulation in blood and moves towards the liver. Once the triglycerides are in the circulation, carried by VLDL, the cholesterol ester from HDL is transferred to the VLDL, and the triglycerides leave the VLDL and replace the cholesterol ester in HDL. The newly formed VLDL is exchanged for the cholesterol ester in LDL, forming small, dense, LDL. The bidirectional transfer of triglyceride leads to elevated triglyceride levels. The removal of the cholesterol ester from HDL makes HDL very susceptible to breakdown and excretion resulting in a reduction of HDL levels (Fig. 2). Further elucidation of mechanistic studies with cell and knockout mice models might add value to the study results.

## Conclusion

From the obtained results it is confirmed that there exists a functional link between fatty acid desaturase gene polymorphism and lipid profile ststus in type-2 diabetes mellitus. Further research on how fatty acid desaturases exert its effect on triglyceride levels needs to be enumerated. This will provide a wide insight into the effect of fatty acid desaturases on human health and disease. Further this might lead to the development of fatty acid desaturase activity based personalized therapeutic strategies.
